# Genetics of growth resilience- and uniformity-related indicators derived from sparse body weight data in Angus–Brangus cattle under (sub)tropical conditions

**DOI:** 10.1186/s12711-026-01073-6

**Published:** 2026-08-02

**Authors:** Mário L. Santana, Luiz F. Brito, Diercles F. Cardoso, Mario L. Piccoli, Annaiza B. Bignardi

**Affiliations:** 1 Grupo de Melhoramento Animal de Mato Grosso (GMAT), Instituto de Ciências Agrárias e Tecnológicas, Universidade Federal de Rondonópolis, Rondonópolis, MT 78735-901 Brazil; 2 Department of Animal Sciences, Purdue University, West Lafayette, IN 47907 USA; 3 GenSys Consultores Associados S/S, Porto Alegre, RS 90460-060 Brazil

## Abstract

**Background:**

Resilience is an important breeding objective in livestock, reflecting the capacity of animals to maintain their performance or recover quickly from short-term environmental perturbations. The derivation of resilience indicators requires longitudinal measurements of key traits. In beef cattle, the limited availability of frequently recorded variables restricts the development of resilience-related indicators. This limitation is particularly relevant in (sub)tropical regions of the Southern Hemisphere, where animals are exposed to greater environmental variability and indicators of growth stability could therefore be especially valuable. Therefore, the main objective of this study was to derive resilience- and uniformity-related indicators from sparse body weight records in a Brazilian Angus–Brangus population, estimate their genetic parameters, and assess their associations with other economically relevant traits.

**Results:**

Six indicators potentially related to growth resilience and uniformity were derived from observed and expected body weight trajectories, representing complementary biological dimensions: area under the curve, mean absolute deviation, logarithm of the mean of squares, logarithm of variance, relative maximum drawdown, and recovery slope index. A cubic quantile regression provided the best fit for reference growth trajectories compared with nonlinear models. All indicators were heritable, with direct and maternal heritability estimates ranging from 0.029 to 0.240 and from 0.024 to 0.071 for logarithm of variance and recovery slope index, respectively. Relative maximum drawdown (0.24) and recovery slope index (0.18) showed the highest heritabilities, while logarithm of the mean of squares (0.039) and logarithm of variance (0.029) had the lowest ones. The additive genetic coefficient of variation was highest for recovery slope index, indicating substantial genetic variability. Genetic and phenotypic correlations formed biologically consistent clusters, with area under the curve–mean absolute deviation and logarithm of the mean of squares–logarithm of variance closely related (> 0.90), and relative maximum drawdown–recovery slope index strongly associated (− 0.93) but distinct from the others. Partial genetic correlations, accounting for average growth potential, were modest but consistently favorable across 16 growth, carcass, adaptation, temperament, and reproduction traits. Mean absolute deviation showed favorable associations with key traits, including pre-weaning gain (− 0.39) and yearling conformation score (− 0.27), as well as with tick count (0.16). In contrast, the recovery slope index showed favorable correlations with pre- and post-weaning gain (0.39 and 0.32), backfat thickness (0.30), and age at first calving (− 0.15). Overall, the recovery slope index combined high heritability, favorable genetic relationships with key traits, and low redundancy with other indicators.

**Conclusions:**

Resilience- and uniformity-related indicators derived from sparse body-weight records of beef cattle are heritable with substantial additive genetic variance. Recovery slope index and mean absolute deviation are the most promising indicators for breeding applications. Recovery slope index captured variation in growth recovery not represented by other indicators and showed strong potential for improving growth recovery ability, while mean absolute deviation provided a broad and consistent profile of favorable associations with growth, carcass, adaptation, temperament, and reproduction traits. Overall, this study establishes a framework for quantifying resilience- and uniformity-related variation in growth trajectories of Angus–Brangus beef cattle, thereby contributing to productivity and sustainability under challenging environmental conditions.

**Supplementary Information:**

The online version contains supplementary material available at 10.1186/s12711-026-01073-6.

## Background

Resilience in livestock species can be defined as the capacity of individual animals to withstand environmental or management-related disturbances with minimal and transient impacts on performance [1, 2]. In the current context of climate change, water scarcity, soil degradation, disease pressure, and deforestation [3, 4], genetic selection for more resilient animals may contribute to more efficient and sustainable livestock production. Beyond ensuring productivity under adverse conditions, resilience has also been associated with improved animal health and welfare, as more resilient animals tend to recover faster from environmental perturbations and maintain more stable performance trajectories [5, 6]. In countries such as Brazil, where cattle production occurs across highly heterogeneous environments, improving stability and recovery capacity in animal performance becomes even more critical.

Selection for improved resilience in farm animals can be achieved using indicators derived from longitudinal phenotypes over time, such as body weight, feed intake, behavior, or health records, which reflect the animal’s response and recovery to environmental perturbations [5–7]. A common approach is to project the expected performance trajectory of each animal in the absence of perturbations and compare it with the corresponding observed performance. The deviations between observed and expected values at the individual level are then used to derive widely applied metrics, such as the logarithm of variance, skewness, and autocorrelation of the resulting residual series [8–10]. Such indicators provide a quantitative basis for assessing the stability of performance over time and across environments. Lower or less persistent deviations indicate greater stability, whereas larger or more irregular deviations reflect reduced stability. Based on these indicators, genetic parameters and estimated breeding values can be obtained, thereby enabling selection for resilience in the same manner as for any other trait in breeding programs. Despite the importance of resilience as a breeding objective, most studies to date have focused on dairy cattle [6, 8], pigs [10, 11], sheep [12, 13], and chickens [14], with limited research in beef cattle. Rodrigues et al. [15, 16] applied growth models to derive resilience-related indicators in beef heifers and reported heritability estimates ranging from 0.08 to 0.42, along with favorable genetic correlations with reproduction and longevity traits (e.g., − 0.28 to − 0.37).

The derivation of resilience-related indicators generally draws on longitudinal data collected at multiple points throughout an animal’s life. In beef cattle, however, such longitudinal records are often limited, even for commonly measured traits such as body weight. In many herds, animals are weighed only at birth, weaning, and yearling. Consequently, the limited number of records can hinder the derivation of reliable indicators of resilience-related responses [17]. This constraint highlights the need for methodological approaches capable of extracting meaningful information on growth stability, recovery, and uniformity even from sparse or irregular datasets. In this context, the main objectives of this study were to: (1) derive novel resilience- and uniformity-related indicators from sparse records of the growth trajectory of Brazilian Angus–Brangus beef cattle; (2) estimate their (co)variance components and genetic parameters; and (3) assess genetic and phenotypic associations among indicators and between indicators and other economically relevant traits routinely evaluated in the breeding program.

## Methods

### Dataset

The database used in the present study was obtained from the “NATURA” breeding program (https://gensys.com.br/) and included birth weight (BW, kg), weaning weight (WW, kg), and yearling weight (YW, kg) records of 135,885 Brazilian Angus–Brangus (Angus × Nellore) cattle. Of the total records analyzed, 30.1% corresponded to animals from pure Angus sires and dams, 37.6% to 5/8 Angus sires with 5/8 Angus dams, 7.2% to 5/8 Angus sires with 1/2 Angus dams, and 1.1% to animals recognized as Ultrablack, i.e., progeny of pure Angus sires and 5/8 Angus dams or vice versa. Together, these breed compositions accounted for 76.1% of all body weight records. The animals were born between 1986 and 2023 in herds located across different regions of Brazil. Further details on herd distribution are provided in Alves et al. [18]. The animals were maintained on pasture, with or without feed supplementation during the dry season. Pastures were predominantly composed of *Brachiaria brizantha* in most farms, with exception of those located in the Pampa region (Southern Brazil) where native grasslands were more commonly used. All farms provided mineral supplementation, and some of them also supplied protein supplements. Weaning took place when calves were approximately seven months of age, and YW was recorded at around 18 months of age. The distribution of body weight records according to age is shown in Additional File 1 Fig. S1.

## Contemporary group, datasets, and quality control of phenotypic information

Only animals with at least two body weight records were considered in the analyses. Contemporary groups (CG) were defined by combining information on herd at birth, weaning, and yearling, sex, and year and season of birth. Calving season was classified as dry (April to September, 68% of the records) or rainy (October to March, 32% of the records). To ensure consistency in CG assignment across animals with different numbers of body weight records, CG were deliberately defined as longitudinal units spanning the period from birth to yearling, rather than separately by growth phase. Specifically, when no herd change occurred between birth and yearling, a single herd identifier was used to define the CG for the entire growth period. Under this definition, animals from the same herd were assigned to the same CG regardless of whether they had two (BW and WW) or three (BW, WW, and YW) body weight records. For animals that changed herds between weaning and yearling, both herd identifiers were included in the CG code, irrespective of whether a YW record was available. No animal changed herds between birth and weaning.

Only records from calves born to cows aged between 2 and 13 years were retained [3,921 records excluded]. Age at weaning ranged from 115 to 295 days (average 218 days), and age at yearling ranged from 365 to 640 days (average 527 days) [13,197 records excluded]. The definition of yearling age was relaxed due to its wider distribution across different ages and because our aim was to provide the most accurate description possible of the animals’ growth curve. Two specific quality control procedures were applied with respect to CG and number of records, resulting in two datasets: (1) to describe the reference growth curve of animals and calculate indicators potentially related to growth resilience and uniformity, and (2) to estimate variance components and genetic parameters.

Dataset 1: For the calculation of reliable indicators, repeated measurements of animal performance are required. In the present population, as in many others, body weight was available only at birth, weaning, and yearling. Such sparse records could lead to inaccurate descriptions of individual growth curves [19] and affect the estimation of genetic parameters for resilience indicators [17]. To mitigate this limitation, growth curves were described collectively within each CG, meaning that a single growth curve was projected for each group of animals that experienced approximately the same environmental conditions. Because sex influences growth patterns, it was included in the CG definition, resulting in sex-specific growth trajectories within each environmental context. For this dataset, CG were required to include at least five animals, with a minimum of three records for BW, three for WW, and three for YW [41,521 records excluded]. Under this scenario, calves from multiple-sire mating groups were also considered.

Dataset 2: This dataset was used to estimate variance components and genetic parameters, and therefore stricter criteria were adopted. Only records from animals with known sires and dams were considered, and each animal was required to have BW, WW, and YW records. In addition, each CG was required to include at least ten animals [43,764 records excluded]. In this case, sex was not included in the CG definition because resilience- and uniformity-related indicators had already been derived relative to sex-specific growth trajectories, and sex was subsequently fitted as a separate effect in the genetic model.

For both datasets (1) and (2), all observations were required to fall within four standard deviations above or below the CG mean [595 records excluded]. This threshold, as applied by Poppe et al. [8] was chosen to retain records that may be informative for detecting growth disturbances. A description of the datasets analyzed is presented in Table 1.

**Table 1:** Summary of the data structures used to obtain the phenotypic records of resilience- and uniformity-related indicators (1) and to estimate variance components (2) in an Angus–Brangus cattle population

Item	Dataset 1	Dataset 2
Animals in the pedigree file, n	–	154,421
Sires in the pedigree file, n	–	3,872
Dams in the pedigree file, n	–	68,832
Animals with records, n	135,885	92,121
Sires with progeny records, n	–	3,019
Dams with progeny records, n	–	53,551
Genotyped animals with records, n	–	7,342
Genotyped sires, n	–	793
Genotyped dams, n	–	1,059
Genotyped sires with own records, n	–	283
Genotyped dams with own records, n	–	682
Progeny records from genotyped sires, n	–	51,326
Progeny records from genotyped dams, n	–	5,261
Total number of records, n	397,923	276,363
Birth weight (BW) records, n	126,404	92,121
Weaning weight (WW) records, n	135,850	92,121
Yearling weight (YW) records, n	135,669	92,121
Contemporary groups, n	2,446	1,873
Ages with weight measurements per CG, n	50.48 (9 to 205)*	48.31 (12 to 179)*
Mean (standard deviation) BW, kg	33.53 (5.17)	33.73 (5.13)
Mean (standard deviation) WW, kg	190.97 (41.82)	196.53 (41.92)
Mean (standard deviation) YW, kg	318.95 (75.55)	329.17 (77.35)

*Mean (minimum and maximum)

## Genomic datasets and quality control

A total of 15.985 animals (4.303 Angus and 11.682 crossbreds) were genotyped as part of ongoing genotyping activity of the NATURA breeding program, comprising several different single nucleotide polymorphism (SNP) panels, including NeoGen^®^ GeneSeek Genomic Profiler (GGP; 50 K, 100 K and 150k), Zoetis^®^ i50K and HD50k, and Genetic Visions ST^®^ VMT. SNPs located on non-autosomal chromosomes or with unknown or ambiguous genome positional information of any panel were not kept for further analyses. A combined set of 138,964 autosomal SNPs, comprising markers from the GGP100k and VMT panels, was defined as the standard panel for imputation. This approach is routinely applied in the genetic evaluation of the breeding program and was previously validated in animals that were genotyped with both panels (*n* = 350). Therefore, missing and unknown genotypes were imputed using the FImpute software [20]. Quality control considered the following criteria for removing information: SNPs and samples with a call rate lower than 0.90, minor allele frequency lower than 0.01, and extreme deviation from Hardy–Weinberg equilibrium (*P* < 10⁻⁵). After this procedure, 126,035 informative SNPs for 11,570 animals were retained for subsequent analyses.

## Quantile-based modelling of growth trajectories

In beef cattle, growth curves from birth to yearling can be used to describe the expected body weight trajectory of animals under comparable environmental conditions. When animals experience environmental or management disturbances, their observed weights may temporarily deviate from this trajectory. The magnitude and temporal structure of these deviations provide information on how animals differ in growth stability and recovery capacity relative to a reference trajectory, and can therefore be used to derive indicators related to resilience. A major challenge, however, is that disturbances are rarely recorded and may differ among individuals, making it difficult to define a reference trajectory representing growth under relatively unperturbed conditions. In addition, the sparse nature of beef cattle growth records precludes the reliable estimation of an individual unperturbed trajectory. As mentioned previously, to address this limitation, we modelled collective growth patterns within CG using the upper quantile of the growth distribution to represent animals expressing their growth potential under similar conditions and therefore less affected by perturbations. Deviations between observed individual records and this reference trajectory were then quantified and summarized into indicators reflecting resilience-related responses, describing the magnitude, variability, and recovery dynamics of growth perturbations. Additional File 2 Fig. S2 illustrates the deviations of two animals selected to represent contrasting levels of deviation magnitude.

To model animal growth, we employed the quantile polynomial regression method, which has been successfully applied in resilience studies [8, 21]. Using dataset 1, the polynomial order was defined through careful visual inspection of growth patterns across dozens of CG, and by evaluating the adequacy of model fitting across all available data. We attempted, with parsimony, to avoid model over-parameterization, given that the growth period under study was relatively short (up to 640 days) and weight records within each CG were temporally sparse. Under these conditions, a cubic quantile regression model was identified as an appropriate representation. A quantile of 0.7 was adopted, following the rationale of Poppe et al. [8] when deriving dairy cattle resilience based on lactation curve analyses. Indeed, choosing a quantile greater than 0.5 is generally considered a more suitable option to approximate the potential growth trajectory in the absence of disturbances. The quantile regression model can be expressed as:

1 $$\:{W}_{cr}={\beta\:}_{c0}+{\beta\:}_{c1}{t}_{cr}+{\beta\:}_{c2}{t}_{cr}^{2}+{\beta\:}_{c3}{t}_{cr}^{3}+{e}_{cr}$$

where, $\:{W}_{cr}$ was the observed body weight for record * r* within contemporary group * c*, $\:{t}_{cr}$ was the corresponding age in days, $\beta_{c0}$, $\beta_{c1}$, $\beta_{c2}$, and $\beta_{c3}$ were contemporary-group-specific regression coefficients, and $\:{e}_{cr}$ was the residual term.

In addition to the polynomial model, three classical sigmoidal nonlinear functions were evaluated to describe animal growth: the Gompertz, Brody, and von Bertalanffy models. These models were chosen based on the studies of Forni et al. [22] and Rodrigues et al. [15], which analyzed growth curves of *Bos indicus* and *Bos taurus* cattle under Brazilian conditions. Because nonlinear models for growth curve parameters are conditional on mean body weight, we applied a 0.7 quantile regression to these functions. This approach, similar to that of Nascimento et al. [23], was used to describe the growth trajectory of animals from each contemporary group in the absence of disturbances. For all nonlinear models, $\:{t}_{cr}$ denoted age in days, $\:{A}_{c}$ was the asymptotic weight, $\:{b}_{c}$ was a constant related to the initial conditions of growth, and $\:{k}_{c}$ was the maturation rate parameter for contemporary group * c*. The observed body weight records were modeled as:

2 $$W_{{cr}} = A_{c} \exp \left[ { - b_{c} \exp \left( { - k_{c} t_{{cr}} } \right)} \right] + e_{{cr}} ,{\text{ }}\left[ {{\mathrm{Gompertz}}} \right] $$

3 $$ W_{{cr}} = A_{c} \left[ {1 - b_{c} \exp \left( { - k_{c} t_{{cr}} } \right)} \right] + e_{{cr}} ,\quad \left[ {{\mathrm{Brody}}} \right] $$

4 $$ W_{{cr}} = A_{c} \left[ {1 - b_{c} \exp \left( { - k_{c} t_{{cr}} } \right)} \right]^{3} + e_{{cr}} ,\quad \left[ {{\mathrm{von}}\;{\mathrm{Bertalanffy}}} \right] $$

Polynomial and nonlinear quantile regression models (rq and nlrq functions) were fitted using the quantreg R package [24]. To compare the goodness of fit among the alternative growth models, we calculated the pseudo-R² metric proposed by Koenker and Machado [25], which is an analogue of the classical coefficient of determination of least squares regression. For a given quantile $\:\tau\:$, the statistic was defined as:

5 $$\:{R}^{1}\left(\tau\:\right)=1-V\left(\tau\:\right)/{V}_{0}\left(\tau\:\right)$$

where $\:V\left(\tau\:\right)$ denoted the weighted sum of absolute residuals from the fitted model and $\:{V}_{0}\left(\tau\:\right)$ the corresponding value from a model including only an intercept. This measure provides a local assessment of goodness of fit at quantile $\:\tau\:$, allowing model comparison under the quantile regression framework. Because a growth curve was fitted for each CG, a pseudo-R² was obtained for each group, and both the median and the standard deviation of this statistic were subsequently calculated.

## Development of resilience- and uniformity-related indicators

To characterize growth stability and recovery patterns, six indicators were calculated from the deviations of individual body weights relative to the predicted growth curve within the corresponding CG. These indicators can be grouped into three complementary biological categories related to resilience and uniformity. The first category, magnitude of deviation, quantifies the absolute extent to which an animal diverges from the predicted growth trajectory of its CG, and includes both the area under the curve (AUC) and the mean absolute deviation (MAD) of deviations. The second category focusing on variability describes the fluctuations of deviations around the predicted curve and includes the logarithm of mean squares (LMS) and the logarithm of variance (LNVAR). The third group focuses on directional vulnerability and recovery by capturing the most pronounced single collapse together with the animal’s ability to recover between consecutive measurements and includes the relative maximum drawdown (MDD) and the recovery slope index (RSI).

The MAD, LMS, and LNVAR capture distinct aspects of the overall oscillation and dispersion of deviations. Lower values of these indicators denote greater stability of individual performance relative to the contemporary-group reference curve and, therefore, may reflect more favorable resilience- and uniformity-related patterns. Conversely, higher values indicate larger fluctuations and greater variability around the expected curve, consistent with reduced growth stability. MAD was calculated as:

6 $$\:{\mathrm{M}\mathrm{A}\mathrm{D}}_{j}=\frac{1}{{n}_{j}}\sum\:_{i=1}^{{n}_{j}}\left|{d}_{i,j}\right|$$

where, $\:{d}_{i,j}$ denotes the deviation between observed and expected body weight (kg) of animal * j* at age $\:{t}_{i,j}$ (days); $\:{t}_{i,j}$ was the age corresponding to the $\:{i}^{\mathrm{th}}$ weight record of animal * j*; and $\:{n}_{j}$ was the total number of body weight records available for that animal. The LMS was defined as:

7 $$\:{\mathrm{L}\mathrm{M}\mathrm{S}}_{j}=\mathrm{l}\mathrm{o}\mathrm{g}\left(\frac{1}{{n}_{j}}\sum\:_{i=1}^{{n}_{j}}{d}_{i,j}^{2}\right)$$

and the LNVAR was computed as:

8 $$\:{\mathrm{L}\mathrm{N}\mathrm{V}\mathrm{A}\mathrm{R}}_{j}=\mathrm{l}\mathrm{o}\mathrm{g}\left[\frac{1}{{n}_{j}-1}\sum\:_{i=1}^{{n}_{j}}{\left({d}_{i,j}-\bar{{d}}_{j}\right)}^{2}\right]$$

where $\bar{{d}}_{j}$ was the mean deviation of animal * j*. The AUC was computed using the trapezoidal rule, which approximates the integral of deviations over time by summing the areas of trapezoids formed between successive observations. Because our interest was in the total magnitude of variation, trapezoids were calculated separately, and the absolute values of their areas were then summed. In this way, animals with an AUC close to zero showed greater stability relative to the contemporary-group reference curve, whereas higher AUC values indicated larger cumulative deviations over the growth trajectory. The formula adopted here can be expressed as:

9 $$\:AU{C}_{j}={\varSigma\:}_{i=1}^{{n}_{j}-1}\left|\frac{{d}_{i,j}+{d}_{i+1,j}}{2}\left({t}_{i+1,j}-{t}_{i,j}\right)\right|$$

where, the notation is as previously defined. The original AUC values were divided by 1,000 to avoid numerical scaling issues in the genetic analyses.

The MDD metric, a concept widely used in finance to quantify the largest peak-to-trough decline of an asset [26], was here adapted to characterize abrupt decreases in growth trajectory deviations. It captures the most pronounced single collapse in the sequence of deviations across the growth trajectory. Lower MDD values indicate greater stability relative to the contemporary-group reference curve and, therefore, more favorable resilience- and uniformity-related patterns, whereas higher values indicate greater vulnerability to abrupt drops in the deviation trajectory. MDD can be expressed as:

10 $$\:MD{D}_{j}=\frac{max\left(0,{d}_{1,j}-{d}_{2,j},{d}_{1,j}-{d}_{3,j},{d}_{2,j}-{d}_{3,j}\right)}{max\left(\left|{d}_{1,j}\right|,\left|{d}_{2,j}\right|,\left|{d}_{3,j}\right|\right)+{10}^{-6}}$$

where, $\:{d}_{1,j}$, $\:{d}_{2,j}$, and $\:{d}_{3,j}$ denoted the deviations of animal * j* at birth, weaning, and yearling, respectively. The numerator corresponded to the largest non-negative drop observed across pairs of deviations, with potential increases set to zero, while the denominator rescaled this value by the maximum absolute deviation observed for the animal. A small constant (10^− 6^) was included to avoid division by zero.

**Fig. 1:**
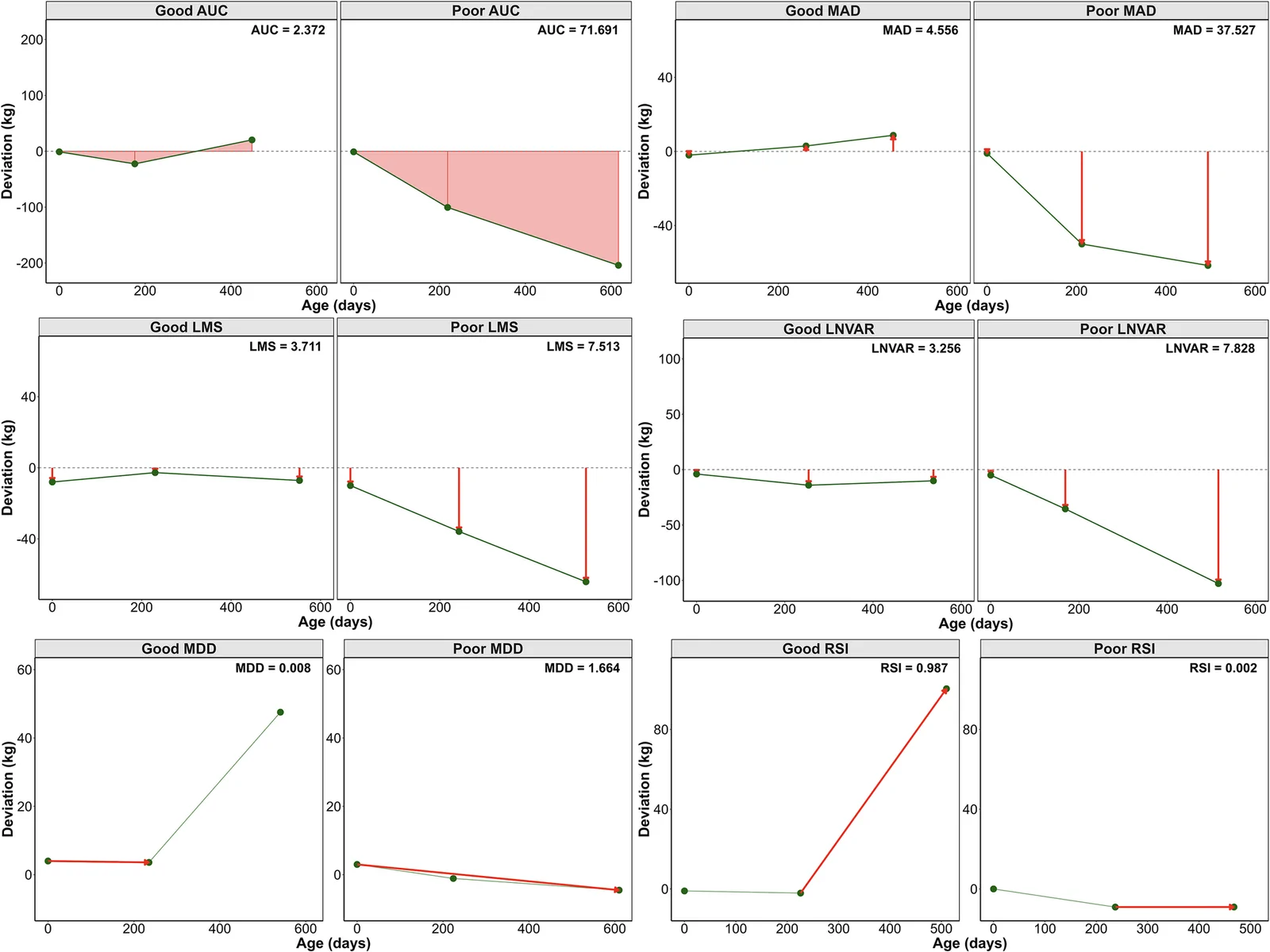
Schematic representation of the derivation of growth resilience- and uniformity-related indicators from deviations around the expected body weight trajectory. The solid line represents the expected growth trajectory estimated by quantile regression, whereas points correspond to the body weight records observed. Vertical distances between observed and expected values define the deviations over time. These deviations are subsequently summarized into indicators. AUC: area under the curve, MAD: mean absolute deviation, LMS: logarithm of mean of squares, LNVAR: logarithm of variance, MDD: relative maximum drawdown, RSI: recovery slope index, BW: birth weight, WW: weaning weight, YW: yearling weight

As a proxy for engineering resilience [27] and based on recovery rate metrics previously studied in dairy cattle [6, 28], the RSI was proposed to capture the ability of animals to recover following disturbances in their growth trajectory. Higher RSI values indicate a greater proportion of positive slopes in the deviation trajectory relative to the total magnitude of change across consecutive records, suggesting a more favorable growth recovery pattern. In contrast, lower RSI values denote reduced resilience, as they are associated with predominantly negative or unstable deviations. Formally, the RSI for animal * j* was defined as:

11 $$\:\mathrm{RS}{\mathrm{I}}_{\mathrm{j}}=\frac{{\sum\:}_{i=1}^{{n}_{j}-1}max(0,{s}_{i,j})}{{\sum\:}_{i=1}^{{n}_{j}-1}\left|{s}_{i,j}\right|+{10}^{-6}}$$

where, $\:{s}_{i,j}=\frac{{d}_{i+1,j}-{d}_{i,j}}{{t}_{i+1,j}-{t}_{i,j}}$ denoted the slope between consecutive records. The numerator retained only positive slopes (i.e., recoveries), while negative slopes were set to zero. The denominator corresponded to the sum of absolute slopes across all intervals, thereby normalizing the numerator by the total magnitude of change and producing a scale-free proportion. A small constant (10^− 6^) was included to prevent division by zero. Descriptive statistics of the resilience- and uniformity-related indicators in dataset 1 are presented in Table 2. In Fig. 1, animals with contrasting values (good and poor) for each indicator defined above were randomly sampled to illustrate how these metrics capture deviations in performance relative to the expected growth within their corresponding CG.

**Table 2:** Descriptive statistics of the growth resilience- and uniformity-related indicators in Brazilian Angus–Brangus cattle

Trait	AUC	MAD	LMS	LNVAR	MDD	RSI
N	92,121	92,121	92,121	92,121	92,121	92,121
Mean	10.43	19.63	5.91	5.52	0.81	0.34
Standard deviation	8.71	13.88	1.55	1.53	0.43	0.36
Minimum	0.00	0.02	− 6.73	− 7.11	0.00	0.00
Maximum	104.44	160.72	10.86	10.79	2.00	1.00
Coefficient of variation	83.49	70.68	26.29	27.99	53.04	104.69

AUC: area under the curve, MAD: mean absolute deviation, LMS: logarithm of mean of squares, LNVAR: logarithm of variance, MDD: relative maximum drawdown, RSI: recovery slope index

### Genetic analyses

The (co)variance components for body weight records and resilience- and uniformity-related indicators were obtained using the dataset 2 and two-trait animal models. The basic model for all traits was similar to that applied in official genetic evaluations [29] and included the effects of CG (as defined above) and the following covariates: age of the animal at weaning (linear effect; only for WW) and yearling (linear effect; only for YW), sex, and age of the dam at calving (linear and quadratic effects), individual and maternal breed composition (percentage of Angus breed as a linear effect), and individual and maternal heterozygosity (linear effects). Therefore, variation in additive, dominance, and recombination effects among different crossbred types was not explicitly modelled. This model can be expressed in matrix notation as follows:

12 $$\:\mathbf{y}\:=\:\mathbf{X}\boldsymbol{\upbeta\:}\:+\:{\mathbf{Z}}_{\mathbf{d}}\:\mathbf{d}\:+\:{\mathbf{Z}}_{\mathbf{m}}\:\mathbf{m}\:+\:\mathbf{e}$$

where, $\:\mathbf{y}$ was the vector of observations for each trait; $\:\boldsymbol{\upbeta\:}$ was the vector with solutions for the systematic effects and covariates; $\:\mathbf{d}$ and $\:\mathbf{m}$ were the vectors with the solutions for the direct and maternal additive genetic effect, assumed as $\:\mathbf{d}\:\sim\:N(0,\:\mathbf{H}{\sigma\:}_{d}^{2})$ and $\:\mathbf{m}\:\sim\:N(0,\:\mathbf{H}{\sigma\:}_{m}^{2})$, where $\:\mathbf{H}$ was the relationship matrix that combines pedigree and genomic information, and $\:{\sigma\:}_{d}^{2}$ and $\:{\sigma\:}_{m}^{2}$ were the direct and maternal additive genetic variances, respectively; $\:\mathbf{e}$ was the vector of residual effects, assumed as $\:\mathbf{e}\:\sim\:N(0,\:\mathbf{I}{\sigma\:}_{e}^{2})$, where $\:\mathbf{I}$ was an identity matrix and $\:{\sigma\:}_{e}^{2}$ was the residual variance; $\:\mathbf{X}$, $\:{\mathbf{Z}}_{\mathbf{d}}$, and $\:{\mathbf{Z}}_{\mathbf{m}}$ were the incidence matrices for the systematic, direct additive genetic, and maternal additive genetic effects, respectively. The assumptions of the additive genetic effects were:

13 $$ \left[ {\begin{array}{*{20}c} {d_{1} } \\ {d_{2} } \\ {m_{1} } \\ {m_{2} } \\ \end{array} } \right]\sim N\left( {\begin{array}{*{20}c} {0,~H \otimes } \\ \end{array} \left[ {\begin{array}{*{20}c} {\sigma _{{d1}}^{2} } & {\sigma _{{d1,d2}} } & {\sigma _{{d1,m1}} } & {\sigma _{{d1,m2}} } \\ {\sigma _{{d1,d2}} } & {\sigma _{{d2}}^{2} } & {\sigma _{{d2,m1}} } & {\sigma _{{d2,m2}} } \\ {\sigma _{{d1,m1}} } & {\sigma _{{d2,m1}} } & {\sigma _{{m1}}^{2} } & {\sigma _{{m1,m2}} } \\ {\sigma _{{d1,m2}} } & {\sigma _{{d2,m2}} } & {\sigma _{{m1,m2}} } & {\sigma _{{m2}}^{2} } \\ \end{array} } \right]} \right) $$

where, $\:{\sigma\:}_{d1}^{2}$, $\:{\sigma\:}_{d2}^{2}$, $\:{\sigma\:}_{m1}^{2}$, and $\:{\sigma\:}_{m2}^{2}$ were the direct and maternal additive genetic variances of traits 1 and 2, respectively; $\:{\sigma\:}_{d1,d2}$ and $\:{\sigma\:}_{m1,m2}$ were the direct and maternal additive genetic covariances between trait 1 and 2, respectively; and the terms $\:{\sigma\:}_{d-,m-}$ represented the genetic covariances between direct and maternal effects for traits 1 and 2. For the YW model, the maternal additive genetic effect was not included. Given that 60.4% of dams had only one progeny record, the maternal permanent environmental effect was disregarded across all traits. The analyses were performed following the single-step genomic BLUP framework, based on a Bayesian approach implemented in the GIBBSF90 + program [30]. Each chain comprised 150,000 samples, with a burn-in of 40,000 samples and a thinning interval of 25, resulting in 4,400 posterior samples per bivariate analysis. Because bivariate analyses were employed, the posterior samples for each trait were consistently used across all analyses to estimate the genetic parameters of interest. For instance, AUC was represented in eight distinct analyses involving different traits (BW, WW, YW, MAD, LMS, LNVAR, MDD, RSI), and therefore the genetic parameters such as heritability were estimated based on 35,200 posterior samples (4,400 × 8). Convergence was monitored by examining trace plots to assess the mixing pattern of the chains.

Direct and maternal heritability estimates were calculated as:

14 $$\:{h}_{d}^{2}=\frac{{\sigma\:}_{d}^{2}}{{\sigma\:}_{d}^{2}\:+\:{\sigma\:}_{m}^{2}\:+\:{\sigma\:}_{d,m}\:+{\:\sigma\:}_{e}^{2}}$$

15 $$\:{h}_{m}^{2}=\frac{{\sigma\:}_{m}^{2}}{{\sigma\:}_{d}^{2}\:+{\:\sigma\:}_{m}^{2}\:+{\:\sigma\:}_{d,m}\:+{\:\sigma\:}_{e}^{2}}$$

The covariance between direct and maternal additive genetic effects, expressed as a proportion of the phenotypic variance ($\:{c}_{dm}$), was used to calculate the direct–maternal additive genetic correlation as:

16 $$\:{r}_{dm}=\frac{{c}_{dm}}{\sqrt{{h}_{d}^{2}\times\:{h}_{m}^{2}}}$$

The genetic coefficient of variation (GCV) was calculated as $\:\sqrt{{\sigma\:}_{d}^{2}}/\mu\:$ (i.e., the square root of the direct genetic variance divided by the phenotypic mean of the animals in the data) providing a scale-free measure of the relative amount of genetic variation available. For LNVAR and LMS, the GCV was expressed as $\:{\sigma\:}_{a}$, because the log-transformation of the variance implies an exponential model in which the division by $\:\mu\:$ is redundant [8, 31]. The GCV for maternal genetic effects was calculated analogously to that for direct genetic effects.

## Correlations of resilience- and uniformity-related indicators with growth, carcass, adaptation, and reproduction traits

The growth potential of animals may exhibit genetic relationships not only with the resilience- and uniformity-related indicators proposed herein but also with several growth, carcass, adaptation, and reproduction traits routinely included in the official Brazilian Angus–Brangus genetic evaluation program. To assess the genetic associations between these indicators independently of the animals’ average growth potential, we computed partial genetic correlations. First, the average direct genetic merit for growth from birth to yearling (GROWd) was defined as the mean of standardized direct genomic estimated breeding values (GEBV) for BW, WW, and YW, and the average maternal genetic merit for growth to weaning (GROWm) as the mean of standardized maternal GEBV for BW and WW. Second, individual GEBV were retrieved from the official genetic evaluation of the present population, covering the following 16 traits, each described with its original unit of measurement and the desired direction of expected progeny differences: pre-weaning weight gain (PreWG, kg, higher); post-weaning weight gain (PosWG, kg, higher); visual score of body conformation at weaning (WC, 1–5, higher) and yearling (YC, 1–5, higher); visual score of finishing precocity at weaning (WP, 1–5, higher) and yearling (YP, 1–5, higher); visual score of musculature at weaning (WM, 1–5, higher) and yearling (YM, 1–5, higher); *Longissimus dorsi* muscle area at yearling (LMA, cm², higher); backfat thickness at yearling (BFT, mm, higher); tick count (TICK, count, lower); visual score of coat at weaning (WCOAT, 1–3, lower) and yearling (YCOAT, 1–3, lower); temperament score at yearling (TEMP, 1–5, lower); scrotal circumference at yearling (SC, cm, higher); and age at first calving (AFC, days, lower).

Similarly to the procedure applied by Chen et al. [9], the approximate genetic correlations between resilience- and uniformity-related indicators and the traits of interest were estimated using Pearson correlation coefficients, considering only animals with GEBV accuracy > 0.45 for direct effects and > 0.35 for maternal effects. The partial genetic correlations were calculated as described by Poppe et al. [8]:

17 $$\:{r}_{xy,z}\hspace{0.25em}=\hspace{0.25em}\frac{\hspace{0.17em}{r}_{xy}-{r}_{xz}\hspace{0.17em}{r}_{yz}\hspace{0.17em}}{\sqrt{\left(1-{r}_{xz}^{2}\right)}\sqrt{\left(1-{r}_{yz}^{2}\right)}}$$

where, * x* was the resilience- and uniformity-related indicator (direct or maternal GEBV); * y* was one of the 16 traits listed above; and * z* was either GROWd or GROWm. It was not possible to obtain standard errors for the partial genetic correlations.

## Results

### Quantile-based modelling of growth trajectories

**Fig. 2:**
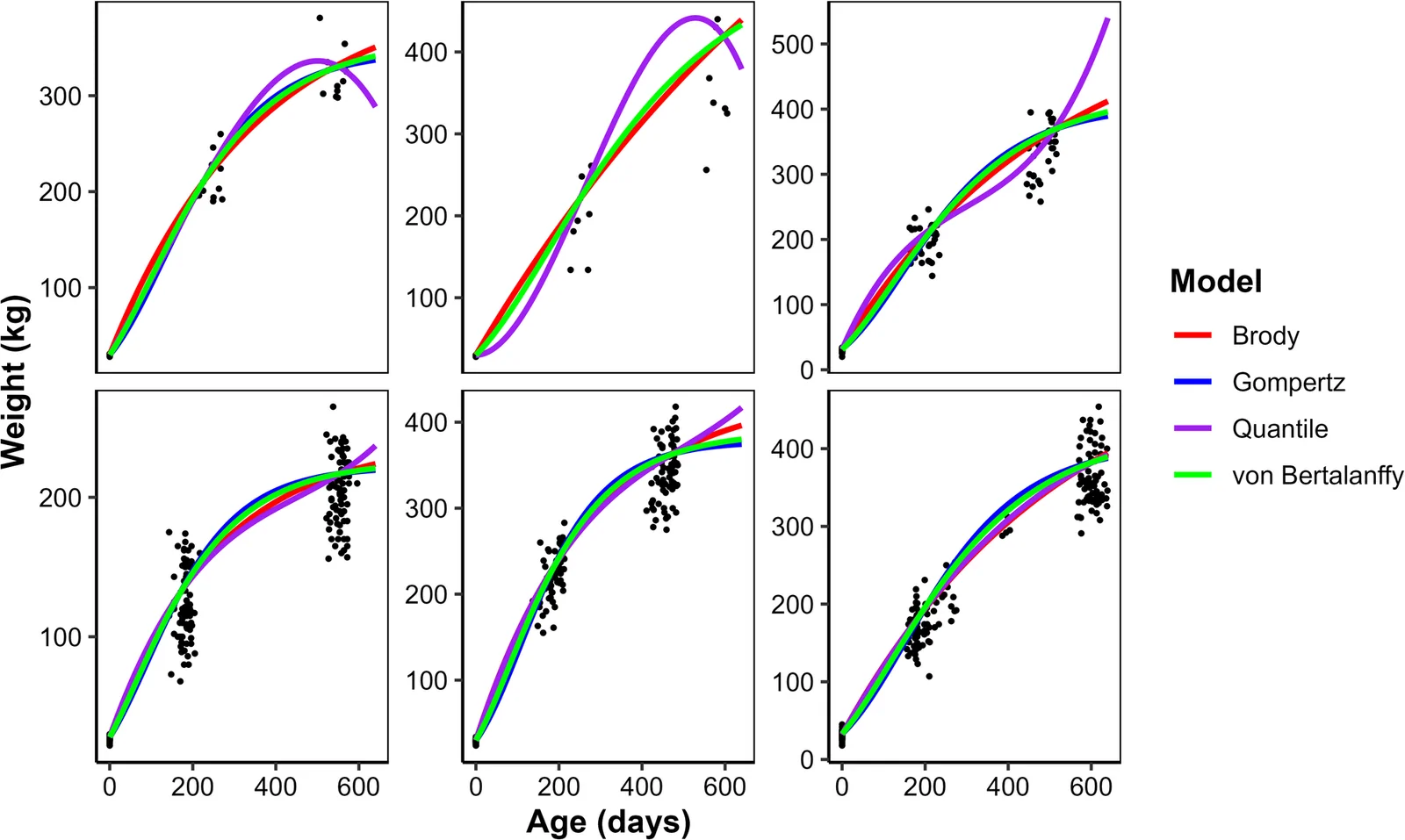
Comparison of quantile-based growth models cubic polynomial (Quantile), von Bertalanffy, Brody, and Gompertz showing expected body weight rajectories for six randomly sampled contemporary groups of Brazilian Angus–Brangus cattle. Each panel represents a distinct contemporary group. Colored lines correspond to the fitted trajectories from each model, and black points represent observed body weight records used for model fitting

Using dataset 1, the overall goodness of fit of the cubic polynomial, von Bertalanffy, Brody, and Gompertz models was compared. The median (standard deviation) of the pseudo-R^2^ values for these models were 0.842 (0.058), 0.835 (0.060), 0.832 (0.061), and 0.830 (0.060), respectively. Accordingly, the cubic polynomial quantile regression model was considered the most appropriate for describing the reference growth trajectories within each CG. Based on this model, deviations of individual body weights from the predicted growth curve were subsequently calculated. Figure 2 presents the expected growth curves from each model for six CG randomly sampled from the data.

### Heritability, genetic coefficient of variation, and direct–maternal correlations

The posterior means of direct heritability estimates for the indicators were low to moderate, ranging from 0.029 to 0.24 (Table 3). The LMS and LNVAR indicators, both related to fluctuations of deviations around the predicted growth curve, exhibited the lowest heritability estimates. In contrast, the MDD and RSI indicators, associated with directional vulnerability and recovery, showed the highest heritability estimates obtained in this study. The AUC and MAD indicators, which quantify the absolute extent to which an animal diverges from the expected growth trajectory, presented low heritability values. Maternal heritability estimates followed approximately the same pattern of magnitude as those observed for direct genetic effects of each indicator. Posterior means of maternal heritability ranged from 0.024 for LNVAR to 0.058 for AUC. Among body weight traits, BW showed the highest posterior mean of direct heritability (0.319), whereas WW presented the highest posterior mean of maternal heritability (0.181).

**Table 3:** Genetic parameters for resilience- and uniformity-related indicators and body weights of Brazilian Angus–Brangus cattle

Parameter	Trait	Mean (SD)	HPD95%
$\:{h}_{d}^{2}$	AUC	0.106 (0.010)	0.089 to 0.127
	MAD	0.101 (0.009)	0.085 to 0.120
	LMS	0.039 (0.007)	0.026 to 0.052
	LNVAR	0.029 (0.007)	0.018 to 0.041
	MDD	0.161 (0.015)	0.137 to 0.192
	RSI	0.240 (0.012)	0.217 to 0.264
	BW	0.319 (0.014)	0.294 to 0.347
	WW	0.223 (0.012)	0.200 to 0.246
	YW	0.289 (0.008)	0.273 to 0.305
$\:{h}_{m}^{2}$	AUC	0.058 (0.007)	0.045 to 0.073
	MAD	0.047 (0.006)	0.036 to 0.060
	LMS	0.029 (0.005)	0.021 to 0.038
	LNVAR	0.024 (0.004)	0.017 to 0.033
	MDD	0.051 (0.009)	0.036 to 0.070
	RSI	0.071 (0.008)	0.055 to 0.087
	BW	0.103 (0.009)	0.088 to 0.121
	WW	0.181 (0.011)	0.158 to 0.203
$\:{GCV}_{d}$	AUC	0.268	–
	MAD	0.219	–
	LMS	0.353	–
	LNVAR	0.294	–
	MDD	0.200	–
	RSI	0.519	–
$\:{GCV}_{m}$	AUC	0.200	–
	MAD	0.152	–
	LMS	0.333	–
	LNVAR	0.302	–
	MDD	0.117	–
	RSI	0.296	–
$\:{r}_{dm}$	AUC	− 0.238 (0.075)	− 0.375 to − 0.077
	MAD	− 0.180 (0.080)	− 0.324 to − 0.015
	LMS	− 0.184 (0.121)	− 0.394 to 0.075
	LNVAR	− 0.327 (0.130)	− 0.549 to − 0.042
	MDD	− 0.513 (0.084)	− 0.639 to − 0.348
	RSI	− 0.294 (0.050)	− 0.383 to − 0.183
	BW	− 0.487 (0.032)	− 0.547 to − 0.423
	WW	− 0.418 (0.042)	− 0.494 to − 0.328

AUC: area under the curve, MAD: mean absolute deviation, LMS: logarithm of mean of squares, LNVAR: logarithm of variance, MDD: relative maximum drawdown, RSI: recovery slope index, BW: birth weight, WW: weaning weight, YW: yearling weight, SD: standard deviation; HPD95% = 95% highest posterior density intervals; $\:{h}_{d}^{2}$ = direct heritability, $\:{h}_{m}^{2}$ = maternal heritability; $\:{r}_{dm}$= correlation between direct and maternal genetic effects; * GCV* = genetic coefficients of variation; * d* = direct genetic effect; * m* = maternal genetic effect

The RSI presented the highest GCV values for both direct (0.519) and maternal effects (0.296) (Table 3). The LMS and LNVAR indicators also showed relatively high GCV values, with estimates close to or exceeding 0.300 for both direct and maternal effects. In contrast, MDD exhibited the lowest GCV values for direct (0.200) and maternal effects (0.117).

Posterior means of direct–maternal correlations were consistently negative for all indicators as well as for BW and WW (Table 3). MDD showed the strongest posterior mean estimate of this parameter (− 0.513), whereas MAD presented the weakest one (− 0.180). BW and WW exhibited negative direct–maternal correlations of moderate magnitude, with posterior means of − 0.487 and − 0.418, respectively.

### Genetic and phenotypic correlations between resilience- and uniformity-related indicators and body weights

**Fig. 3:**
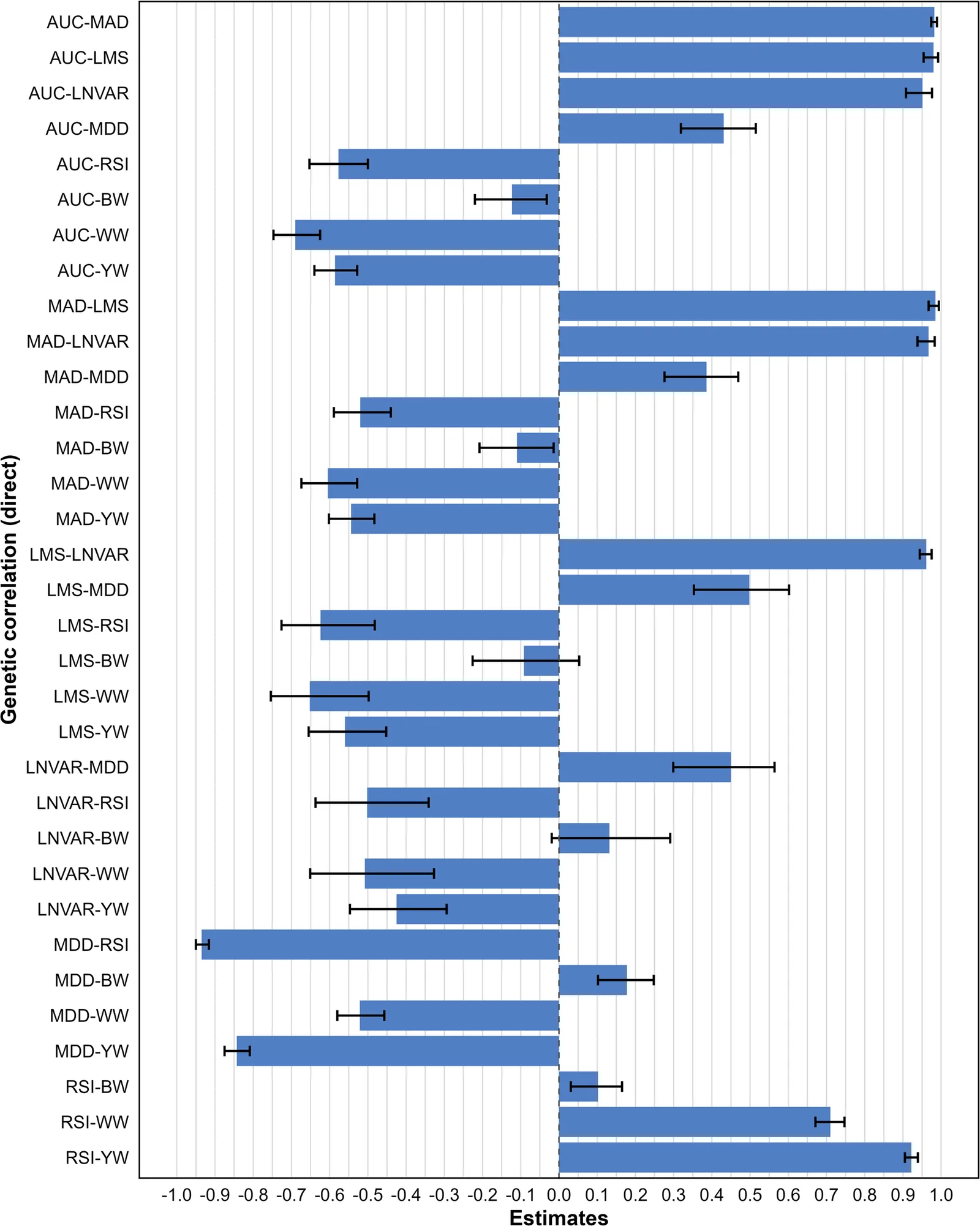
Posterior means (bars) and 95% highest posterior density intervals (lines) of the estimates of direct genetic correlation among resilience- and uniformity-related indicators and body weights of Brazilian Angus–Brangus cattle. AUC: area under the curve, MAD: mean absolute deviation, LMS: logarithm of mean of squares, LNVAR: logarithm of variance, MDD: relative maximum drawdown, RSI: recovery slope index, BW: birth weight, WW: weaning weight, YW: yearling weight

In general, the genetic correlations obtained among resilience- and uniformity-related indicators and between indicators and body weights were favorable (Fig. 3). Indicators representing the same biological category showed strong genetic relationships, with posterior means of magnitude exceeding 0.90. For example, the direct genetic correlations between AUC–MAD, LMS–LNVAR, and MDD–RSI were 0.982, 0.961, and − 0.935, respectively. AUC and MAD exhibited more moderate correlations with indicators related to directional vulnerability and recovery (MDD and RSI). In contrast, their associations with LMS and LNVAR were very close to unity. Overall, MDD displayed weaker genetic relationships with the remaining indicators (AUC, MAD, LMS, and LNVAR), whereas LMS showed the strongest direct relationships with other indicators, with posterior means ranging from 0.498 to 0.985.

When resilience- and uniformity-related indicators were compared with body weights, the overall pattern of direct genetic correlations was consistent and favorable, although magnitudes varied depending on the trait. On average, LMS and LNVAR exhibited the weakest direct correlations with weights, whereas MDD and RSI were the most strongly correlated indicators. For BW, the weakest estimates were accompanied by comparatively wide highest density intervals, ranging from − 0.092 (LMS–BW) to 0.178 (MDD–BW). For WW, genetic correlations with resilience- and uniformity-related indicators were of moderate magnitude, with values spanning from − 0.508 (LNVAR–WW) to − 0.690 (AUC–WW). Correlations with YW were slightly stronger, ranging from − 0.425 (LNVAR–YW) to 0.922 (RSI–YW).

**Fig. 4:**
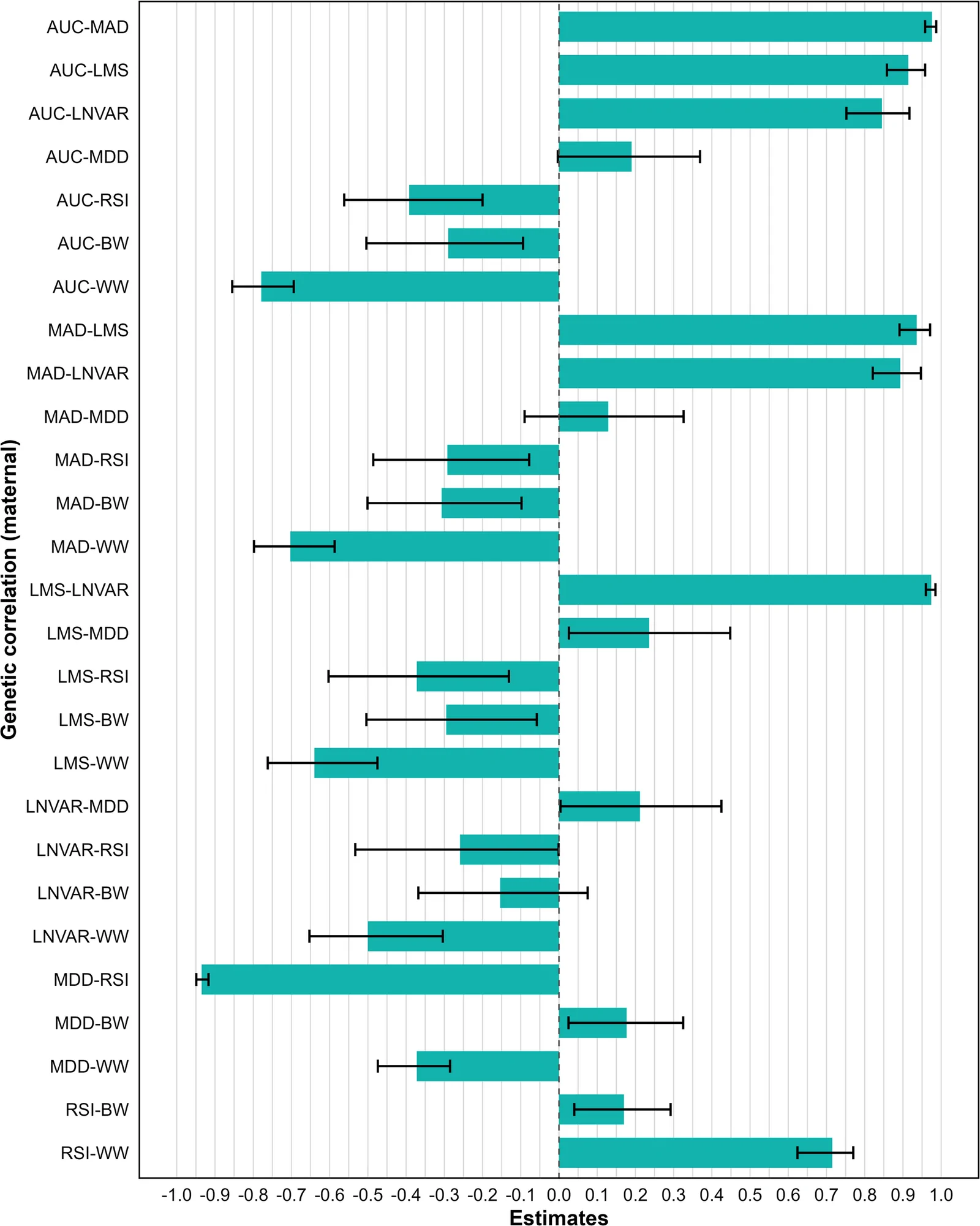
Posterior means (bars) and 95% highest posterior density intervals (lines) of the estimates of maternal genetic correlation among resilience- and uniformity-related indicators and body weights of Brazilian Angus–Brangus cattle. AUC: area under the curve, MAD: mean absolute deviation, LMS: logarithm of mean of squares, LNVAR: logarithm of variance, MDD: relative maximum drawdown, RSI: recovery slope index, BW: birth weight, WW: weaning weight

Maternal genetic correlations among the indicators were favorable and tended to be of lower magnitudes than their direct counterparts, generally preserving the same sign (Fig. 4). Maternal correlations were particularly weaker for MDD and RSI with the other indicators. For example, whereas the direct genetic correlation between LMS and MDD was 0.498, the maternal correlation was 0.236. For LNVAR–RSI, the posterior mean of the direct genetic correlation was − 0.502 and the maternal estimate was − 0.259. For BW, maternal genetic correlations with the resilience- and uniformity-related indicators were generally more pronounced than their direct counterparts. For example, AUC–BW had a direct genetic correlation of − 0.123 and a maternal correlation of − 0.290. For LNVAR–BW, the direct genetic correlation was 0.132 and the maternal estimate was − 0.154. In contrast, maternal correlations for MDD–BW and RSI–BW were close to their direct counterparts. For WW, the maternal pattern was similar to that observed for direct estimates. Maternal genetic correlations for AUC–WW (− 0.779) and MAD–WW (− 0.703) were slightly more negative than the direct ones, whereas the maternal posterior mean for MDD–WW was weaker (− 0.372) than the direct estimate (− 0.521).

**Fig. 5:**
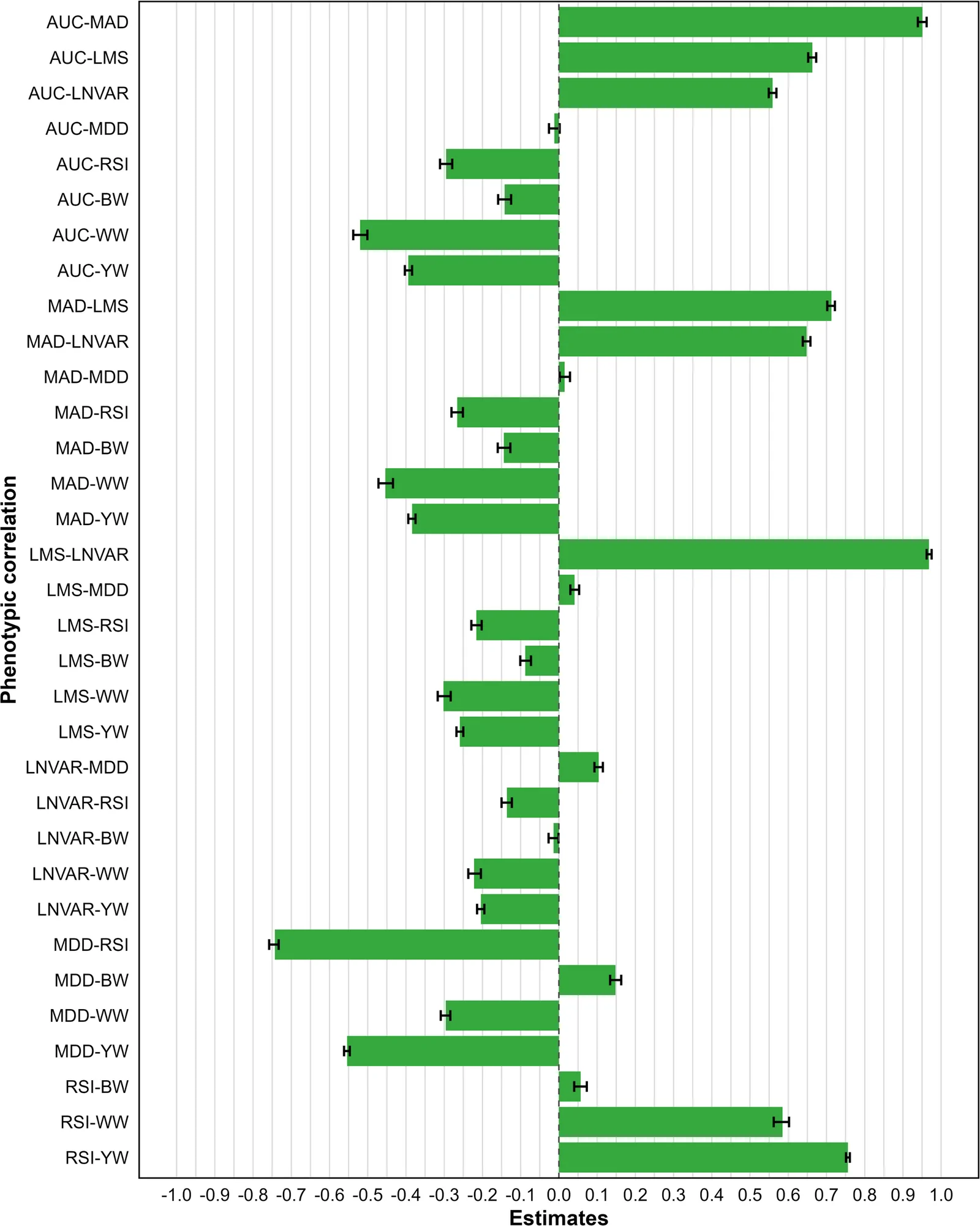
Posterior means (bars) and 95% highest posterior density intervals (lines) of the estimates of phenotypic correlation among resilience- and uniformity-related indicators and body weights of Brazilian Angus–Brangus cattle. AUC: area under the curve, MAD: mean absolute deviation, LMS: logarithm of mean of squares, LNVAR: logarithm of variance, MDD: relative maximum drawdown, RSI: recovery slope index, BW: birth weight, WW: weaning weight, YW: yearling weight

In general, phenotypic correlations (Fig. 5) showed smaller magnitudes than their direct additive genetic counterparts and largely preserved the sign, with two near-zero exceptions: AUC–MDD (− 0.012 phenotypic vs. 0.431 direct additive genetic) and LNVAR–BW (− 0.014 vs. 0.132). Phenotypic correlations were strong among indicators belonging to the same biological category. However, several indicator pairs showed lower phenotypic than genetic correlations, such as AUC–LMS (0.663 vs. 0.980), AUC–LNVAR (0.559 vs. 0.951), MAD–LMS (0.713 vs. 0.985), and MAD–LNVAR (0.648 vs. 0.967). Phenotypic correlations with BW were uniformly weak, ranging from − 0.144 (MAD–BW) to 0.148 (MDD–BW). For WW and YW, phenotypic correlations with the indicators were stronger, ranging from − 0.520 (AUC–WW) to 0.756 (RSI–YW).

### Genetic correlations between resilience- and uniformity-related indicators and growth, carcass, adaptation, temperament, and reproduction traits

**Fig. 6:**
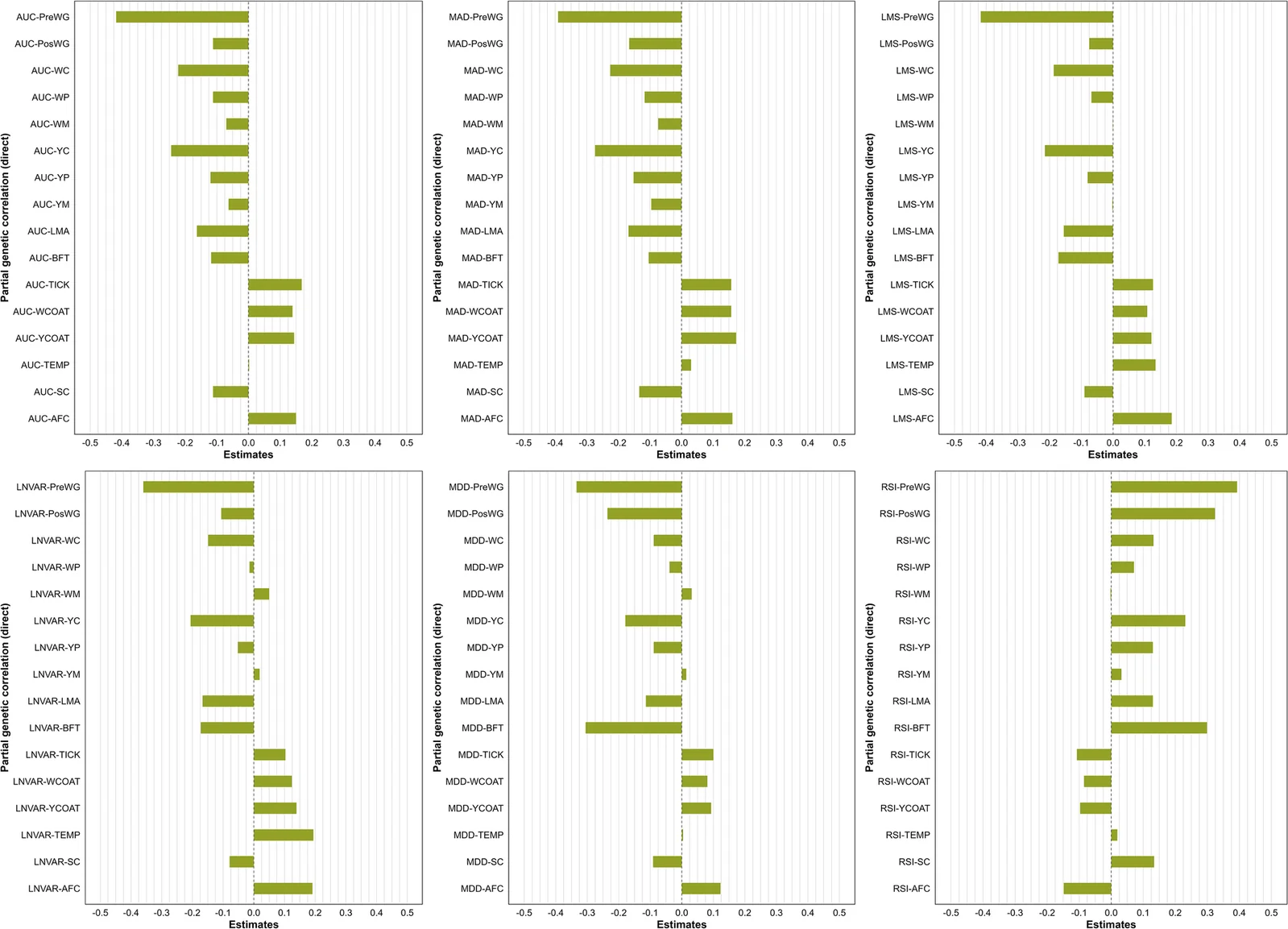
Partial genetic correlations between the direct genetic effects of resilience- and uniformity-related indicators and the 16 traits officially evaluated in the Brazilian Angus–Brangus breeding program. AUC: area under the curve, MAD: mean absolute deviation, LMS: logarithm of mean of squares, LNVAR: logarithm of variance, MDD: relative maximum drawdown, RSI: recovery slope index, PreWG: pre-weaning weight gain, PosWG: post-weaning weight gain, WC: visual score of body conformation at weaning, YC: visual score of body conformation at yearling, WP: visual score of finishing precocity at weaning, YP: visual score of finishing precocity at yearling, WM: visual score of musculature at weaning, YM: visual score of musculature at yearling, LMA: longissimus muscle area at yearling, BFT: backfat thickness at yearling, TICK: tick count; WCOAT: visual score of coat at weaning, YCOAT: visual score of coat at yearling, TEMP: temperament score at yearling, SC: scrotal circumference at yearling, AFC: age at first calving

Overall, all resilience- and uniformity-related indicators considered in this study showed moderate and favorable partial direct genetic correlations with the traits included in the official genetic evaluations of the Brazilian Angus–Brangus breeding program (Fig. 6). AUC (lower values are desirable) showed stronger favorable correlations with growth (PreWG, − 0.418), body conformation (WC, − 0.222; YC, − 0.244), and carcass (LMA, − 0.163) traits, with additional favorable associations with PosWG (− 0.112), WP (− 0.112), BFT (− 0.118), and SC (− 0.112). AUC was also favourably correlated with adaptation and reproduction traits (TICK, 0.168; WCOAT, 0.139; YCOAT, 0.144; AFC, 0.150). MAD (lower values are better) presented a highly concordant profile, showing favorable correlations with growth, carcass, and conformation [PreWG (− 0.391), WC (− 0.226), YC (− 0.274), LMA (− 0.168), PosWG (− 0.166), YP (− 0.152), SC (− 0.134)], and with adaptation/reproduction [TICK (0.157), WCOAT (0.157), YCOAT (0.173), and AFC (0.161)].

The LMS indicator (lower values are better), which captures fluctuations of deviations, showed favorable direct genetic correlations with growth (PreWG, − 0.417), body conformation (WC, − 0.187; YC, − 0.215), carcass (LMA, − 0.155; BFT, − 0.172), adaptation and temperament (TICK, 0.126; WCOAT, 0.108; YCOAT, 0.121; TEMP, 0.134), and reproduction (AFC, 0.185) traits. Similarly, LNVAR (lower values are better) exhibited favorable relationships with growth and conformation (PreWG, − 0.360; YC, − 0.206), carcass (LMA, − 0.167; BFT, − 0.173), and adaptation, reproduction, and behavior (TEMP, 0.194; AFC, 0.191; WCOAT, 0.124; YCOAT, 0.139; TICK, 0.103) traits. Small and unfavorable signals were detected only for WM (0.050) and YM (0.019).

Maximum drawdown (lower values are better) exhibited favorable direct correlations across growth and carcass [PreWG (− 0.334), PosWG (− 0.236), YC (− 0.179), LMA (− 0.114), and notably BFT (− 0.305)], with only minimal and unfavorable associations with WM (0.031) and YM (0.014). RSI (higher values are desirable) showed strong favorable correlations with growth and carcass (PreWG, 0.393; PosWG, 0.324; BFT, 0.299) and with conformation (YC, 0.231; WC, 0.132; YP, 0.130; LMA, 0.130; SC, 0.134) traits, alongside favorable associations with adaptation and reproduction (TICK, − 0.107; AFC, − 0.148) traits. Unfavorable correlations were near zero for WM (− 0.002) and TEMP (0.019).

**Fig. 7:**
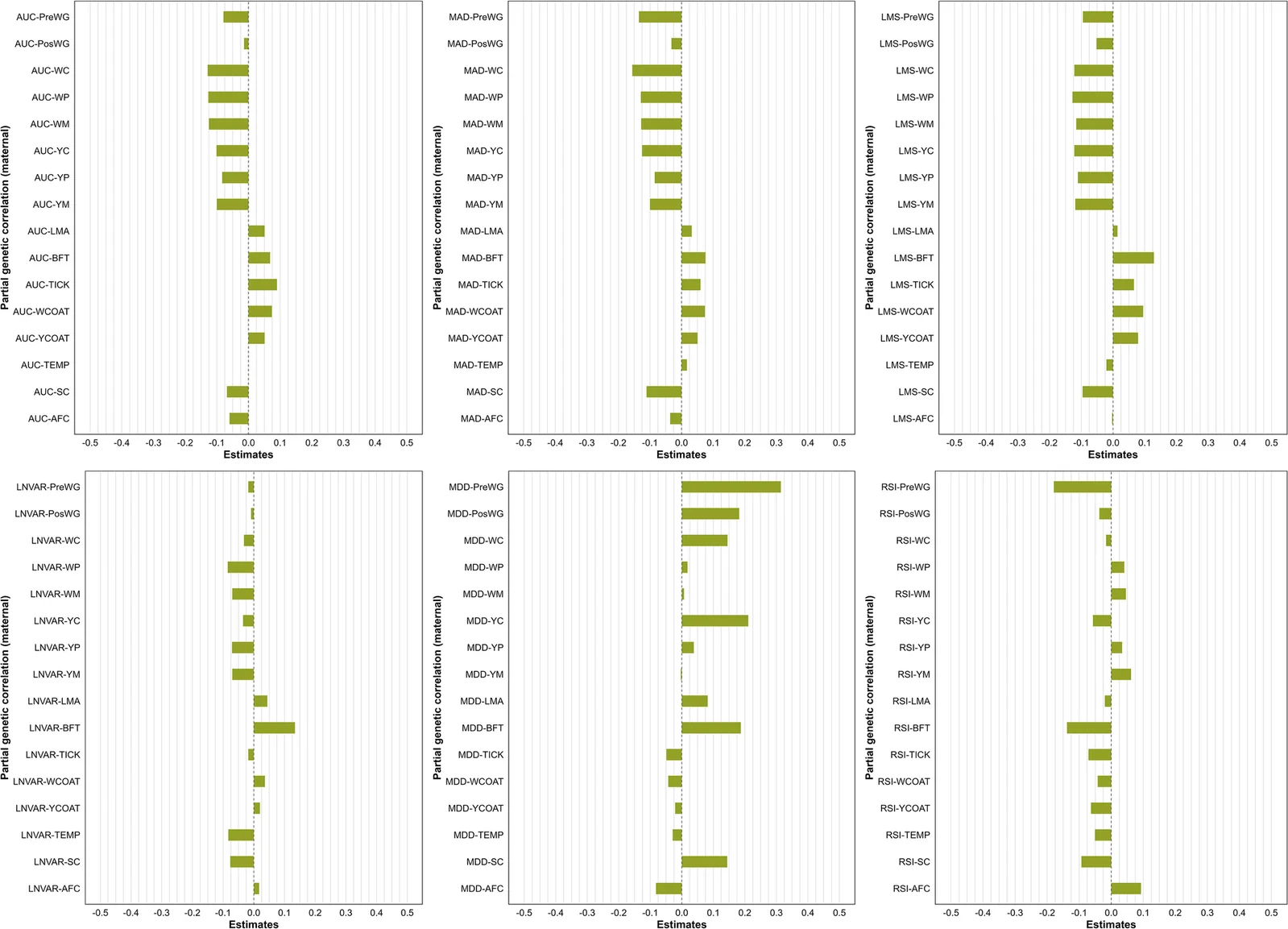
Partial genetic correlations between the maternal genetic effects of resilience- and uniformity-related indicators and the 16 traits officially evaluated in the Brazilian Angus–Brangus breeding program. AUC: area under the curve, MAD: mean absolute deviation, LMS: logarithm of mean of squares, LNVAR: logarithm of variance, MDD: relative maximum drawdown, RSI: recovery slope index, PreWG: pre-weaning weight gain, PosWG: post-weaning weight gain, WC: visual score of body conformation at weaning, YC: visual score of body conformation at yearling, WP: visual score of finishing precocity at weaning, YP: visual score of finishing precocity at yearling, WM: visual score of musculature at weaning, YM: visual score of musculature at yearling, LMA: longissimus muscle area at yearling, BFT: backfat thickness at yearling, TICK: tick count; WCOAT: visual score of coat at weaning, YCOAT: visual score of coat at yearling, TEMP: temperament score at yearling, SC: scrotal circumference at yearling, AFC: age at first calving

The maternal genetic correlations of the indicators were generally weaker than their direct counterparts for growth, conformation, carcass, temperament, and reproduction (Fig. 7). For AUC (lower values are better), more expressive and favorable correlations were observed with body conformation [WC (− 0.129), WP (− 0.127), WM (− 0.125), YC (− 0.101)]; whereas all remaining associations were of low magnitude (|$\:{r}_{xy,z}$| ≤ 0.10), including small unfavorable values for BFT (0.068), LMA (0.051), and AFC (− 0.060). MAD (lower values are better) showed a similar pattern, with favorable negative correlations for growth and conformation [PreWG (− 0.135), WC (− 0.156), WP (− 0.129), WM (− 0.128), YC (− 0.125), SC (− 0.111)], and small unfavorable associations with BFT (0.076), LMA (0.032), and AFC (− 0.036).

The LMS (lower values are better) displayed favorable maternal–direct correlations effects for growth (e.g., PreWG, − 0.095; SC, − 0.096), conformation scores (− 0.111 to − 0.128), and adaptation (TICK, 0.066; WCOAT, 0.095; YCOAT, 0.079) traits, with a small unfavorable association with BFT (0.129). In general, LNVAR (lower values are better) presented weaker correlations across traits, with favorable associations with growth and conformation, and unfavorable associations with TEMP (− 0.083) and BFT (0.134). Several unfavorable correlations were detected for maternal MDD (lower values are better), especially with direct genetic effects of PreWG (0.314), YC (0.211), PosWG (0.182), BFT (0.187), WC (0.145), and SC (0.144). Similarly, RSI (higher values are better) showed a series of unfavorable correlations with direct effects of PreWG (− 0.179), BFT (− 0.138), SC (− 0.093), and AFC (0.093).

## Discussion

In this study, we evaluated six potential indicators of resilience and uniformity derived from deviations between individual observed body weight records and expected growth trajectories defined at the contemporary group level in Brazilian Angus–Brangus cattle. The indicators evaluated were selected based on prior literature [8, 15, 17] and new proposals designed to capture complementary aspects of resilience and uniformity: magnitude of deviation (AUC, MAD), variability (LMS, LNVAR), directional vulnerability (MDD), and recovery (RSI) (Fig. 1).

To describe the growth trajectory in the absence of disturbances, we fitted a quantile regression at τ = 0.70 using a cubic polynomial, alongside the widely used nonlinear functions of Gompertz, Brody, and von Bertalanffy. Quantile regression provides a suitable alternative for representing upper-envelope patterns of performance under putatively undisturbed conditions [8, 17, 21]. Within the observed age range and at τ = 0.70, the cubic quantile polynomial resulted in a more flexible local approximation than the parametric nonlinear alternatives, which impose stricter shape constraints related to asymptotes and inflection points. This was reflected in consistently higher pseudo-R² values across CG. The τ-specific reference trajectories did not consistently display sigmoidal curvature, which was consistent with the better empirical fit obtained with the cubic specification under these data conditions (Fig. 2). Moreover, field datasets of beef cattle growth frequently present irregular measurement intervals and distributions with thin tails at very young or older ages. Under the data configuration of the present Angus–Brangus population, cubic polynomials provided a better fit to the three dense age clusters (birth, weaning, and yearling), which did not consistently cover the asymptotic phase. It is well known that the asymptotic growth phase in Zebu and Angus cattle occurs at later ages than those considered here [32, 33]. In contrast, the nonlinear functions evaluated rely more heavily on adequate data density to identify asymptotes and inflection points [22], which may have impaired their fit to our dataset. Similarly, Berghof et al. [14] reported that, when the number of body weight records per individual is limited, deviations tend to be largely absorbed into the parameters of individual growth curves. Although their study included more repeated measures per animal than the present study, the authors highlighted that fitting individual growth curves may still be challenging when longitudinal data are relatively sparse. In this context, our approach differs in that growth trajectories were modelled at the contemporary group level, allowing a more robust estimation of the expected trajectory, while individual deviations were subsequently quantified relative to this reference. Because the expected trajectories were fitted using nonlinear growth functions on the original body weight scale, deviations at later ages naturally tend to be larger in absolute terms than deviations at birth. Although scaling body weight could reduce this age-related difference in residual magnitude, it would also compromise the biological interpretation of the growth curves and their parameters. Given the sparse longitudinal structure of beef cattle data, we therefore considered the use of absolute deviations from biologically meaningful growth trajectories to be the most appropriate approach.

In this study, we fitted a single reference trajectory for each CG, assuming that individuals within the group were exposed to the same macroenvironment, such as temperature, management, and nutrition, which typically affect most animals. However, even under a shared macroenvironment, differences in response to microvariations may persist, and animals exposed to the same challenge can be affected in distinct ways [14]. We considered the collective description of the target trajectory within CG to be a better option than projecting individual curves based on datasets with sparse records per animal. Berghof et al. [34] demonstrated, using longitudinal data from layer chickens, that the genetic correlations between resilience indicators derived from deviations relative to average batch production and those derived from expected individual production were consistently high for LNVAR, moderate for autocorrelation, and low for skewness. Overall, variance components, heritability, maternal environmental effects, and GCV were comparable between the two definitions of expected production, with the exception that skewness was more sensitive to the choice of reference curve, whereas LNVAR and autocorrelation were scarcely affected. In addition, Berghof et al. [14] concluded that using cohort averages to calculate deviations can be useful when the number of observations per individual is low.

This modelling choice also has implications for the biological interpretation of the proposed indicators. Because the reference trajectory was defined at the contemporary-group level, individual deviations quantify not only departures potentially associated with individual responses to environmental perturbations, but also the degree to which each animal diverges from the expected collective performance of its group. In this framework, the temporal structure of deviations from the contemporary-group trajectory reflects within-group individual variability after accounting for the shared macroenvironment. This interpretation is conceptually consistent with studies on genetic heterogeneity of residual variance, in which residuals from models adjusted for systematic effects are used to investigate genetic differences in residual variability as a basis for improving production uniformity [35, 36]. Genotypes that differ in residual variance may differ in their sensitivity to micro-environmental factors, that is, unidentified animal-specific sources of variation that persist even under a common macroenvironment [37, 38]. Because reduced residual variance has been proposed as a route to improve production uniformity and may also be related to resilience-related outcomes, including health and survival [39], the indicators proposed here should be interpreted as resilience- and uniformity-related traits rather than as pure measures of individual perturbation response. This dual interpretation is an inherent feature of indicators derived from group-level reference trajectories, because they simultaneously describe the stability of individual performance relative to an expected trajectory and the degree of phenotypic consistency among animals raised under comparable conditions [37–39].

As mentioned earlier, the body-weight records of the animals evaluated here were collected at only three specific growth stages. It is recognized that measurement frequency can affect the properties of resilience indicators. In a simulation study, Zefreh et al. [17] reported that skewness and autocorrelation were highly sensitive to measurement frequency, whereas AUC and LMS proved to be more robust resilience-related indicators. These results are consistent with those reported by Gorssen et al. [10], who found that skewness and autocorrelation were more sensitive to record frequency, whereas LNVAR and LMS were more robust for evaluating resilience based on variability in body weight and feed trajectories of growing pigs. Based on these findings, skewness and autocorrelation were not considered as resilience indicators in the present study. Berghof et al. [34] further demonstrated that resilience indicators of egg production computed over different time intervals (1-, 2-, or 3-week) were genetically similar, with consistently high correlations across interval lengths. Although shorter intervals were expected to increase heritability by providing more observations, and longer intervals to capture more variation, the results indicated little effect of interval length on the genetic properties of the indicators. Those authors selected indicators based on 1-week intervals due to their lower environmental variance and higher heritability. Consistent with Gorssen et al. [10], genetic correlations across data recording frequencies for body weight in pigs remained high for LNVAR and LMS (0.79 to 0.97), while the standardized version of LNVAR proved to be the most robust (0.96 to 1.00). Zefreh et al. [17] also reported that a larger observation window relative to the perturbation period is required for resilience indicators to correctly discriminate between response types.

In our case, the number of performance records per animal was limited; nevertheless, considerable genetic variation was detected for each indicator, as discussed below. We therefore hypothesize that body weight may provide a reasonable basis for deriving resilience- and uniformity-related indicators, even when measurements are sparse, as it reflects, at least in part, the gradual accumulation of environmental perturbations on performance. Body weights recorded at later ages are therefore expected to incorporate many of the environmental influences experienced throughout development up to the time of evaluation. Unlike milk yield, body weight responds more gradually to environmental factors, with changes typically becoming apparent only after several days [14]. Thus, even with a limited number of records, deviations from the expected trajectory may retain information on cumulative differences in growth stability among animals. In this context, these indicators should be interpreted as operational proxies potentially related to resilience and uniformity, rather than as direct measures of individual responses to environmental challenges. Our findings are consistent with those of Rodrigues et al. [15], who derived resilience indicators for heifer growth using data from animals with an average of approximately 6.5 and a minimum of three records. Berghof et al. [14] also obtained promising resilience indicators from five to eight body-weight records in laying chickens and reported non-negligible heritability estimates.

To further evaluate the impact of sparse recording, we performed an additional analysis using an independent beef cattle dataset with up to 16 body weight records per animal and a degraded version retaining only three records per animal, one within each growth phase (Additional file 3 Table S1 and Additional file 4 Table S2). Spearman correlations between the complete and degraded versions were 0.67, 0.83, 0.84, 0.79, 0.58, and 0.63 for LNVAR, LMS, MAD, AUC, MDD, and RSI, respectively, indicating moderate to high agreement, particularly for LMS, MAD, and AUC. These indicators were expected to be more consistent because they summarize the overall magnitude or dispersion of deviations and are less dependent on identifying a single extreme point. Conversely, MDD and RSI were more sensitive to data reduction because they depend on the maximum observed deviation or subsequent recovery pattern. Thus, although retaining only three records per animal reduces the amount of longitudinal information, the degraded indicators retained a meaningful proportion of the individual ranking and variability captured by indicators derived from more complete growth records.

The estimates of direct and maternal heritability and of GCV obtained for all resilience- and uniformity-related indicators demonstrated that each of them has, to a greater or lesser extent, potential to capture different biological mechanisms underlying growth resilience (Table 3). Therefore, some of these indicators could be incorporated into breeding programs by considering both their heritability coefficients and their genetic correlations with other economically relevant traits [8]. Importantly, unlike average daily gain, which summarizes overall growth rate between two time points, the proposed indicators characterize the magnitude and temporal structure of deviations from the expected growth trajectory. The direct heritability estimates for LMS and LNVAR were the lowest observed in this study. Studies specifically addressing resilience in beef cattle remain scarce, which prevents more comprehensive comparisons of the present results with literature. Recently, Rodrigues et al. [16] reported direct heritability estimates for LNVAR of body-weight deviations from birth to yearling, from birth to the first breeding season, and from birth to the weaning of the first calf of Nellore heifers of 0.37, 0.42, and 0.32, respectively. Rodrigues et al. [15] also reported a direct heritability estimate of 0.38 for LNVAR of growth trajectories in Nellore and Guzerat heifers (Zebu breeds), whereas a much lower estimate of 0.05 was obtained for Caracu heifers (a Taurine composite breed). These results suggest that this trait may be less heritable in taurine breeds, although further confirmation is needed from studies involving different populations. It is important to note that the studies of Rodrigues et al. [15, 16] did not account for maternal genetic effects in the analyses of the indicators, and the methods used to describe projected growth curves were conditional on individual mean body weight. To the best of our knowledge, the present study is the first to estimate maternal genetic parameters for resilience- and uniformity-related indicators in livestock species. In a study with sheep, Smith et al. [40] reported a direct heritability estimate of 0.06 for LNVAR of body weight, while values ranging from 0.089 to 0.121 were obtained by Gorssen et al. [10] for LNVAR of body weight in pigs. Despite the lower heritability values obtained here for LMS and LNVAR, these indicators contained important genetic information, as reflected by their higher GCV and, consequently, reduced random noise compared with other indicators evaluated. This was particularly evident for maternal genetic effects.

Resilience- and uniformity-related indicators based on the magnitude of deviation (AUC and MAD) exhibited higher direct and maternal heritability estimates than those related to fluctuations of deviations (LMS and LNVAR), indicating that they may respond more satisfactorily to selection. The GCV estimates for both direct and maternal effects further reinforce the existence of considerable potential to exploit genetic differences among animals for these indicators. Smith et al. [40] employed resilience indicators based on principles similar to those of AUC and MAD. Those authors reported heritability estimates of 0.06 and 0.18 for the absolute change in deviation and the area between curves during the weaning disturbance in sheep body weight, respectively. Zefreh et al. [17] demonstrated that LMS, LNVAR, and AUC were the most reliable resilience indicators, as they were less likely to rank non-resilient individuals above partially resilient ones and were more robust to changes in measurement frequency.

In general, the MDD and RSI indicators exhibited the highest posterior means of direct and maternal heritability estimates. In addition, RSI showed the largest GCV for both direct and maternal effects among all indicators evaluated. These results suggest substantial scope for genetic change per generation and important expected proportional gains when selecting for greater recovery capacity in the present population. In dairy cattle, Wang et al. [41] reported low heritability estimates, ranging from 0.004 to 0.061, for several resilience indicators based on milk loss (performance loss, number, and duration of perturbation events). In a study with pigs, Gorssen et al. [10] observed a low heritability estimate (0.062) for the lag-one autocorrelation of weight deviations. In contrast, Rodrigues et al. [15] reported heritability estimates for the lag-one autocorrelation of growth of 0.34 ± 0.07 in Guzerat beef cattle, followed by Nellore (0.10 ± 0.03) and Caracu (0.03 ± 0.02). Autocorrelation was not evaluated in our study due to its strong dependence on measurement frequency, as previously discussed. Nevertheless, it has been one of the most studied resilience indicators, as it captures aspects related to the length of the impact of disturbances. Despite the limited number of studies evaluating resilience indicators related to recovery rate and magnitude of performance losses, these indicators appear to indeed exhibit an important genetic component for growth in beef cattle.

Genetic correlations between direct and maternal effects were negative for all resilience- and uniformity-related indicators, as well as for BW and WW (Table 3). This result is consistent with those reported in several studies of body weights in beef cattle [42–44]. The negative estimates obtained for this parameter can be explained mainly by factors related to the amount of pedigree information, the genetic connectedness among animals, and the number of progeny-dam pairs with their own phenotypic records [44–46]. As reported in the Methods section, most dams in the dataset analyzed here had a single progeny record, which may have influenced the estimates of genetic correlation between direct and maternal effects.

The patterns of genetic and phenotypic correlations observed in the present study highlight that resilience- and uniformity-related indicators tend to cluster according to the biological dimension they were designed to represent (Figs. 3 and 4, and 5). Indicators derived from overall variability, such as LMS and LNVAR, were almost perfectly correlated, indicating that they capture essentially the same genetic signals. This result is consistent with Gorssen et al. [10], who reported a genetic correlation of 0.93 between LNVAR and LMS for body weight in pigs. Similarly, AUC and MAD, both reflecting cumulative deviations, exhibited strong associations with LNVAR and LMS but were only moderately related to directional indicators of perturbation and recovery. In a study using feed intake data in pigs, Mancin et al. [47] found genetic correlations ranging from 0.572 to 0.901 between the logarithm of residual variance and the AUC for periods with the largest consecutive negative errors. These authors inferred that such indicators may represent the same genetic trait. In contrast, MDD and RSI, although strongly correlated with each other, showed weaker associations with the other indicators, suggesting that they quantify distinct aspects of resilience not fully explained by measures of variance or cumulative deviation. In dairy cattle, Wang et al. [6] also derived resilience indicators related to performance losses and recovery. Wang et al. [40] emphasized that the extremely high genetic correlations observed among milk loss traits and among recovery traits indicate that these measures essentially capture the same underlying biological processes. As a result, the authors argued that including only one representative trait from each group would likely be sufficient for genetic evaluations of resilience. Notably, resilience and uniformity indicators are not intended to replace conventional growth traits, but to complement them by capturing aspects of perturbation response and recovery dynamics that are not directly reflected in body weight measurements alone. The identification of resilience and uniformity indicators that capture unique biological dimensions of animal performance is a general concern for researchers and breeders [1, 2, 48]. In this regard, a careful evaluation of genetic parameters, such as heritability and correlations of indicators with other economically important traits, is required within each population. To assess the genetic relationships between indicators independently of average growth potential, we calculated partial genetic correlations.

Although partial genetic correlations between the indicators and the 16 traits evaluated in the Brazilian Angus–Brangus population were moderate, they were consistently favorable. These results are in agreement with several previous studies in dairy cattle [5, 6, 9, 49], pigs [21], sheep [12, 40], and beef cattle [15, 16], in which different resilience indicators were also found to exhibit favorable associations with traits related to health, survival, and reproduction. In absolute terms, MAD showed generally stronger and consistently favorable associations with all 16 traits evaluated. The genetic associations of MAD demonstrated that this indicator carries non-negligible information on PreWG and PosWG, body conformation scores, carcass traits, adaptation, and reproduction. Therefore, we suggest this indicator as a promising broad selection criterion for the present population. In addition, from a practical standpoint, MAD can be considered one of the simplest resilience- and uniformity-related metrics to compute.

With regards to the indicators LMS and LNVAR, which capture fluctuations in performance, the direct genetic associations were comparatively more pronounced for temperament, carcass, and reproduction. However, maternal trade-offs were more frequent for LNVAR than for LMS, indicating that LMS may be the more useful indicator. As noted earlier, both LMS and LNVAR exhibited substantial direct and maternal GCV; however, their heritability estimates were lower and less accurate. Taken together, these results limit the practical application of LMS and LNVAR as primary selection criteria in the Angus–Brangus population.

Among all indicators, MDD and RSI were the most strongly and directly related to carcass traits. MDD also exhibited expressive direct genetic correlations with pre- and post-weaning weight gain. In contrast, this indicator showed consistently unfavorable maternal genetic associations with almost all traits evaluated. These findings led us to not recommend its use in the breeding program of the present population. Conversely, RSI, in addition to exhibiting the highest direct and maternal heritability estimates and the largest GCV, contained relevant direct genetic information for pre- and post-weaning growth, carcass, adaptation, and reproduction. The RSI showed undesirable maternal trade-offs, most notably with PreWG and BFT. However, these unfavorable maternal associations were counterbalanced by stronger favorable direct effects. Although RSI and MDD were strongly correlated with each other, their genetic associations with the 16 routinely evaluated traits demonstrated that RSI may provide more useful information on growth recovery dynamics than MDD for potential incorporation into the breeding program. In addition, the relatively lower magnitude of genetic correlations between RSI and the other resilience- and uniformity-related indicators suggests that RSI captures variation in recovery patterns not fully represented by the remaining indicators.

The inclusion of multiple resilience- and uniformity-related indicators in breeding programs is impractical. As suggested by Poppe et al. [8] and Chen et al. [9], a more feasible strategy lies in the development of a composite index that integrates biologically complementary indicators with the greatest potential for sustainable genetic responses. Such an index would allow the capture of distinct dimensions of growth stability, recovery capacity, and uniformity within a single selection criterion, simplifying its implementation in large-scale genetic evaluations. This approach offers a promising pathway to enhance adaptation, health, and reproductive efficiency while minimizing, or even avoiding, undesirable trade-offs with growth. Ultimately, the incorporation of resilience- and uniformity-related traits into breeding objectives is expected not only to improve the capacity of animals to withstand and recover from environmental disturbances but also to support long-term productivity and the sustainability of beef production systems under increasingly challenging environmental conditions. Future studies could validate the proposed indicators through comprehensive phenotyping of animals with GEBVs for these traits, including physiological biomarkers, health records, functional longevity, and through artificial environmental challenges (e.g., induced thermal stress or pathogen infection).

## Conclusions

Resilience- and uniformity-related indicators can be derived from field records of growth trajectories in Brazilian Angus–Brangus beef cattle. Despite the sparse collection of body-weight records, it was possible to capture relevant dimensions of growth stability, dispersion of deviations, directional changes, and recovery patterns through six complementary indicators. The cubic quantile regression model outperformed traditional nonlinear growth functions in describing reference collective trajectories. All indicators analyzed were heritable; however, those related to fluctuations in performance were considerably less heritable, whereas indicators associated with directional vulnerability and recovery showed the highest heritability estimates. Although moderate in magnitude, the indicators exhibited favorable direct genetic correlations with growth, body conformation, carcass, adaptation, and reproduction. In contrast, non-negligible trade-offs were observed for maternal genetic correlations between some indicators and direct effects of economically relevant traits. Based on these findings, MAD and RSI appear to be the most promising indicators in this population, as they allow the enhancement of adaptation and reproduction without compromising growth. Overall, this study establishes a feasible, data-sparse framework to quantify resilience- and uniformity-related variation in growth trajectories of beef cattle, provides the first set of maternal genetic parameters for such indicators, and identifies RSI and MAD as priority traits for incorporation into Brazilian Angus–Brangus breeding objectives.

## Supplementary Information

Below is the link to the electronic supplementary material.

Supplementary Material 1

Supplementary Material 2

Supplementary Material 3

Supplementary Material 4

## Data Availability

The datasets generated and/or analyzed during the current study are not publicly available due to restrictions from Gensys and the Natura breeding program (https://gensys.com.br/sumario/natura/) but can be obtained from gensys.contato@gmail.com upon reasonable request and signing a data-sharing agreement.
